# Safinamide, an inhibitor of monoamine oxidase, modulates the magnitude, gating, and hysteresis of sodium ion current

**DOI:** 10.1186/s40360-024-00739-5

**Published:** 2024-02-08

**Authors:** Te-Yu Hung, Sheng-Nan Wu, Chin-Wei Huang

**Affiliations:** 1 Department of Pediatrics, Chi-Mei Medical Center, Tainan, Taiwan; 2 Department of Physiology, National Cheng Kung University Medical College, Tainan, Taiwan; 3 Institute of Basic Medical Sciences, National Cheng Kung University Medical College, Tainan, Taiwan; 4 School of Medicine, National Sun Yat-sen University, Kaohsiung, Taiwan; 5 Department of Neurology, National Cheng Kung University Hospital, College of Medicine, National Cheng Kung University, Tainan, Taiwan

**Keywords:** Safinamide, Monoamine oxidase B, Voltage-gated sodium current, Window sodium ion current, Persistent sodium ion current, Voltage-dependent hysteresis

## Abstract

**Background:**

Safinamide (SAF), an α-aminoamide derivative and a selective, reversible monoamine oxidase (MAO)-B inhibitor, has both dopaminergic and nondopaminergic (glutamatergic) properties. Several studies have explored the potential of SAF against various neurological disorders; however, to what extent SAF modulates the magnitude, gating, and voltage-dependent hysteresis [Hys_(V)_] of ionic currents remains unknown.

**Methods:**

With the aid of patch-clamp technology, we investigated the effects of SAF on voltage-gated sodium ion (Na_V_) channels in pituitary GH3 cells.

**Results:**

SAF concentration-dependently stimulated the transient (peak) and late (sustained) components of voltage-gated sodium ion current (*I*_Na_) in pituitary GH_3_ cells. The conductance–voltage relationship of transient *I*_Na_ [*I*_Na(T)_] was shifted to more negative potentials with the SAF presence; however, the steady-state inactivation curve of *I*_Na(T)_ was shifted in a rightward direction in its existence. SAF increased the decaying time constant of *I*_Na(T)_ induced by a train of depolarizing stimuli. Notably, subsequent addition of ranolazine or mirogabalin reversed the SAF-induced increase in the decaying time constant. SAF also increased the magnitude of window *I*_Na_ induced by an ascending ramp voltage *V*_ramp_. Furthermore, SAF enhanced the Hys_(V)_ behavior of persistent *I*_Na_ induced by an upright isosceles-triangular *V*_ramp_. Single-channel cell-attached recordings indicated SAF effectively increased the open-state probability of Na_V_ channels. Molecular docking revealed SAF interacts with both MAO and Na_V_ channels.

**Conclusion:**

SAF may interact directly with Na_V_ channels in pituitary neuroendocrine cells, modulating membrane excitability.

**Supplementary Information:**

The online version contains supplementary material available at 10.1186/s40360-024-00739-5.

## Background

Safinamide [SAF; (S)-2-((4-((3-fluorobenzyl)oxy)benzyl)amino)propanamide], an α-aminoamide derivative, is an oral drug used as either an anticonvulsant or an add-on treatment for Parkinson’s disease when a patient is having an “off” episode [1–18]. However, although this compound was formerly investigated as an anticonvulsant, it has not been approved as a standard antiseizure medication in humans. Several studies also reported the efficacy of SAF as an add-on treatment to subthalamic nucleus deep brain stimulation [19, 20]. SAF exerts its effects through various mechanisms, including inhibition of monoamine oxidase (MAO)-B activity [2, 13–15, 21–24].

Other mechanisms may be involved in SAF-mediated modification of the functional activities [21]. SAF has been demonstrated to protect M17 neuronal cells against amyloid-β-induced oxidative stress and senescence [25]. The evidence supports the notion that SAF might lock Na_V_ channels into the inactivated stage to suppress Na^+^ current [21, 26–29]. It also elevates blood pressure; SAF-induced hypertension may be associated with the inhibition of MAO-B activity [15, 23].

Voltage-gated Na^+^ (Na_V_) channels, which constitute whole-cell voltage-gated Na^+^ currents, are essential for the generation, initiation, and propagation of action potentials in electrically excitable membranes. Nine α subunits of Na_V_ channels (Na_V_1.1 – Na_V_1.9) have been discovered across excitable mammalian tissues, including the central and peripheral nervous systems, the endocrine system, skeletal muscle, and the heart [29–32]. Upon brief depolarization, Na_V_ channels undergo a rapid transition from a resting state to an open state and then rapidly return to the inactivated state of the channel. The cumulative inhibition of *I*_Na_ during a train of depolarizing stimuli was demonstrated to affect the electrical behavior of excitable cells [33–36]. The window *I*_Na_ [*I*_Na(W)_] has been reported to be responsible for background Na^+^ conductance and varying firing patterns of action potentials [37–41]. The Hys_(V)_ of persistent *I*_Na_ [*I*_Na(P)_] induced by a triangular ramp voltage (*V*_ramp_) contributes to the electrical behavior [42, 43]. However, the effects of SAF on the magnitude, gating, and Hys_(V)_ behavior of *I*_Na_ remain to be clarified.

In light of the aforementioned observations, in the present study, we investigated the effects of SAF on the magnitude, gating, frequency dependence, and Hys_(V)_ behavior of *I*_Na_—including transient *I*_Na_ (*I*_Na(T)_), late *I*_Na_ (*I*_Na(L)_), *I*_Na(W)_, and *I*_Na(P)_—in electrically excitable cells.

## Methods

### Chemicals, drugs, reagents, and solutions

SAF [PNU-151774E, Xadago, Equfina, and Fce-26743; (S)-2-((4-((3-fluorobenzyl)oxy)benzyl)amino)propenamide, (2S)-2-[[4-[(3-fluorophenyl)methoxy]phenyl]methylamino]propanamide;methanesulfonic acid, C_17_H_19_FN_2_O_2_, https://pubchem.ncbi.nlm.nih.gov/compound/Safinamide], dopamine, serotonin, tetraethylammonium chloride (TEA), and tetrodotoxin (TTX) were purchased from Sigma-Aldrich (Genechain, Kaohsiung, Taiwan). Metofluthrin was obtained from Chung Tai Sing Chemical Industry (Hsinchu, Taiwan), and mirogabalin (MGB) was obtained from Cayman Chemical (Genechain, Kaohsiung, Taiwan). Ham’s F-12 Nutrient Mix, horse serum, fetal calf serum, L-glutamine, and trypsin/ethylenediaminetetraacetic acid (EDTA) were purchased from HyClone (Thermo Fisher, Tainan, Taiwan). All chemicals and reagents were of analytical grade.

The composition of the external or bath solution [4-(2-hydroxyethyl)-1-piperazineethanesulfonic acid (HEPES)-buffered normal Tyrode’s solution] was as follows: 136.5 mM NaCl, 1.8 mM CaCl_2_, 5.4 mM KCl, 0.53 mM MgCl_2_, 5.5 mM glucose, and 5.5 mM HEPES–NaOH (pH 7.4). To measure K^+^ currents (data not shown), we filled a patch pipette with an internal solution comprising 140 mM KCl, 1 mM MgCl_2_, 3 mM adenosine 5′-triphosphate disodium salt, 0.1 mM guanosine 5′-triphosphate disodium salt, 0.1 mM ethylene glycol tetraacetic acid, and 5 mM HEPES–KOH buffer (pH 7.2). To record the whole-cell *I*_Na_, the K^+^ in the internal solution was substituted with Cs^+^, and the pH of the solution was adjusted to 7.2 by adding cesium hydroxide. In the single-channel experiments performed to record Na_V_ currents, the pipette was filled with a Na^+^-rich solution containing 136 mM NaCl, 0.53 mM MgCl_2_, 5.5 mM glucose, and 5.5 mM HEPES–NaOH (pH 7.2). The bath medium was a K^+^-rich solution comprising 130 mM KCl, 10 mM NaCl, 3 mM MgCl_2_, 6 mM glucose, and 10 mM HEPES-KOH (pH 7.4). The solutions and culture media were generally filtered on the day of use by using sterile Acrodisc Syringe Filters containing a 0.2-μm Supor Membrane (Bio-Check; New Taipei City, Taiwan).

### Cell preparations

GH_3_ pituitary tumor cells, acquired from the Bioresources Collection and Research Center (number: 60,015; Hsinchu, Taiwan), were maintained in Ham’s F-12 media containing 15% (v/v) horse serum, 2.5% (v/v) fetal calf serum, and 2 mM L-glutamine. Cells were grown in a monolayer culture at 37 °C in a humidified environment of carbon dioxide/air (1:19) for 5 or 6 days to a confluence of 60–80%. Trypsinization [0.025% trypsin solution (HyClone) containing 0.01 sodium N,N-diethyldithiocarbamate and EDTA] was performed for subculturing. The culture medium was changed every 2 or 3 days; cells were dispersed and passaged every 7–14 days. Experiments were performed after the cells had grown to a confluence of 60–80% (usually 5 or 6 days). The GH_3_ cell line has been a reliable model for studying the molecular biology, pharmacology, and biophysics of electrically excitable cells, including pituitary endocrine cells.

### Electrophysiological measurements

Shortly before experiments, GH_3_ was carefully suspended in normal Tyrode’s solution at room temperature (20–25 °C). A few drops of the suspension containing cell clumps were immediately added to a custom-built chamber on the stage of an inverted Diaphot-200 microscope (Nikon, Tokyo, Japan). Pipettes were pulled from Kimax-51 soft-glass capillaries (#34500-99; Kimble, Vineland, NJ) by using a Narishige PP-830 Vertical Puller (Tokyo, Japan), and their tips were fire-polished using a microforge (MF-83, Narishige). During the measurements, an electrode with a tip resistance of 2–4 MΩ, which was tightly inserted into a holder, was maneuvered using a WR-98 micromanipulator (Narishige). Patch-clamp experiments were performed in the voltage-clamp mode with either cell-attached or whole-cell configuration (rupturing of the membrane patch after GΩ formation) by using a RK-400 Patch-Clamp Amplifier (Bio-Logic, Claix, France) connected to a laptop [36, 44]. Shortly before GΩ formation, potential correction was performed for a liquid junction potential, which developed at the electrode’s tip because of the difference in the compositions of the internal and bath solutions.

### Data collection and recordings

Amplified signals were monitored using an HM-507 oscilloscope (Hameg, East Meadow, NY); the signals were recorded, digitized, and stored online at ≥10 kHz on a laptop (Sony VAIO CS series; Kaohsiung, Taiwan) connected to an Axon Digidata 1440A Digitizer (Molecular Devices) for efficient analog-to-digital and digital-to-analog conversion. Series resistance, always in the range of 6–18 MΩ, was electronically compensated to 80–95%. Voltage-activated currents recorded during whole-cell experiments were stored without leakage correction. The digitizer was operated using pCLAMP (version 10.6; Molecular Devices) on Windows 10 (Microsoft Corporation, Redmond, WA, USA). To ensure digitalization, some recordings were digitally acquired using the PowerLab 2/26 system (AD Instruments; Kuoyang, New Taipei City, Taiwan). During the measurement, the solutions were exchanged through a homemade gravity-driven type of bath perfusion.

### Data analyses

To evaluate the concentration-dependent stimulatory effects of SAF on I_Na(T)_ and I_Na(L)_, I_Na_ was induced using a 30-ms depolarizing pulse (−100 to −10 mV). The amplitude of the current in SAF-treated and untreated cells was measured at the beginning [I_Na(T)_] and end [I_Na(L)_] of the voltage pulse. The duration of the voltage-clamp protocol is 30 msec and the INa displaying rapid activation and inactivation can be measured at the beginning and end of depolarizing pulse from −100 to −10 mV. The I_Na(T)_ of cells treated with 300 μM SAF was defined as 100% and compared with the current values obtained for different SAF concentrations. The concentration at which SAF increased 50% of the current [I_Na(T)_ or I_Na(L)_] amplitude (EC_50_) was determined using a three-parameter logistic model (modified version of the sigmoidal Hill equation) with goodness-of-fit evaluation:

$Percentage\,increase\,\left( \% \right)  = {\raise0.7ex\hbox{${\left\{ {{E_{max}} \times {{\left[ {SAF} \right]}^{{n_H}}}} \right\}}$} \!\mathord{\left/ {\vphantom {{\left\{ {{E_{max}} \times {{\left[ {SAF} \right]}^{{n_H}}}} \right\}} {\left\{ {EC_{50}^{{n_H}} + {{\left[ {SAF} \right]}^{{n_H}}}} \right\}}}}\right.\kern-\nulldelimiterspace}\!\lower0.7ex\hbox{${\left\{ {EC_{50}^{{n_H}} + {{\left[ {SAF} \right]}^{{n_H}}}} \right\}}$}}$

where EC_50_ is the SAF concentration ([*SAF*]) required for a 50% increase, n_H_ is the Hill slope, and *E*_max_ is the SAF-mediated maximal stimulation of *I*_Na(T)_ or *I*_Na(L)_.

The sigmoidal relationship between V_ramp_-induced *I*_Na(W)_ and the upsloping V_ramp_ (nonlinear current–voltage relationship) was investigated and fitted with the Boltzmann function as follows:

$${\raise0.7ex\hbox{$I$} \!\mathord{\left/ {\vphantom {I {{I_{max}}}}}\right.\kern-\nulldelimiterspace}\!\lower0.7ex\hbox{${{I_{max}}}$}} = {\raise0.7ex\hbox{$G$} \!\mathord{\left/ {\vphantom {G {\left\{ {1 + exp\left[ { - \frac{{\left( {V - {V_h}} \right)qF}}{{RT}}} \right]} \right\}}}}\right.\kern-\nulldelimiterspace}\!\lower0.7ex\hbox{${\left\{ {1 + exp\left[ { - \frac{{\left( {V - {V_h}} \right)qF}}{{RT}}} \right]} \right\}}$}} \times \left( {V - {E_{Rev}}} \right)$$

where *V* is the membrane potential in millivolts, *E*_rev_ is the reversal potential of *I*_Na_, *G* is the *I*_Na_ conductance in nanosiemens, *I* is the current, *V*_h_ is the voltage at which half-maximal activation or inactivation of the current occurs, *q* is the apparent gating charge, *F* is Faraday’s constant, *R* is the universal gas constant, and *T* is the absolute temperature.

The free energy ∆*G*_0_ for the gating of *I*_Na(W)_ was determined by assuming a two-state gating model [equilibrium between closed (resting) and open states] of the Na_V_ channel. The ∆*G*_0_ for the activation of *I*_Na(W)_ at 0 mV could be calculated as follows: *q* × *F* × *V*_1/2_ [45, 46]. The standard errors in ∆*G*_0_ (${\sigma _{q{V_{1/2}}}}$) could be calculated as follows:

$${\sigma _{qF{V_{\frac{1}{2}}}}} = F \times \sqrt {V_{\frac{1}{2}}^2\sigma _q^2 + {q^2}\sigma _{{V_{\frac{1}{2}}}}^2}$$

where σ_q_ and σ_V1/2_ represent the standard error in *q* and *V*_1/2_, respectively.

### Recordings and analyses of single-channel NaV currents

Single-channel Na_V_ currents induced by depolarizing pulses ranging from −100 to −10 mV were measured and subsequently analyzed using pCLAMP 10.7. The opening events of the channels were generally evaluated through multi-Gaussian adjustments of the distribution of amplitude across channels. Functional independence between channels was determined by comparing the observed stationary probabilities with the values calculated based on the binomial law. For dwell-time analyses, only a single channel was used in the patch-clamp experiment.

### Curve-fitting approximations and statistical analyses

Linear or nonlinear curve fitting to different data sets was implemented using the least-squares minimization method through various maneuvers, including the Excel-embedded Solver (Microsoft Corporation) and 64-bit OriginPro (OriginLab; Scientific Formosa, Kaohsiung, Taiwan). The averaged results (whole-cell or single-channel data) are presented in terms of the mean ± standard error of the mean; the number of independent samples (n) indicates the number of cells used for experimental data collection. Between-group differences were analyzed using paired or unpaired Student’s t-test. The differences between more than two groups were evaluated through multiple comparisons performed using analysis of variance (ANOVA)-1 or ANOVA-2 with or without repeated-measures analysis, which was followed by a post-hoc Fisher’s least-significant difference test. Statistical significance was set at P < 0.05 (indicated using ^*, **^, or ^+^ in the figures).

## Results

### Effects of SAF on I_Na_ magnitude

**Fig. 1:**
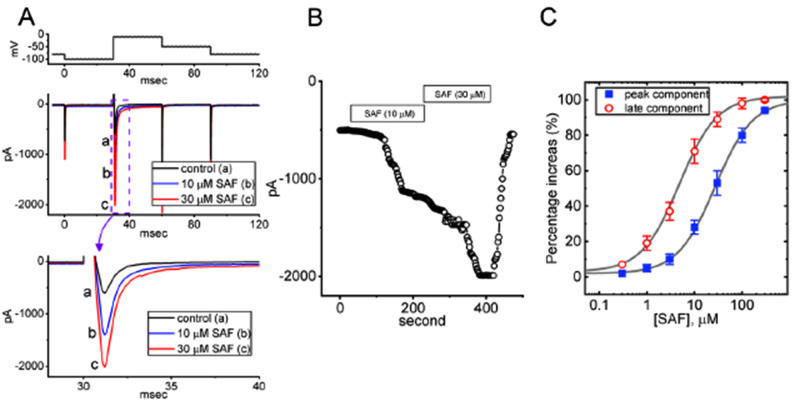
Effect of safinamide (SAF) on the voltage-gated Na^+^ current (*I*_Na_) in pituitary tumor (GH_3_) cells. To record macroscopic currents, calcium ion–free Tyrode’s solution containing 10 mM tetraethylammonium chloride and 0.5 mM cadmium chloride was added to the cells; the recording electrode was filled with solution containing cesium ions. (**A**) Current traces during the control period (a, black; untreated cells) and during exposure to 10 (b, blue) and 30 (c, red) μM SAF. The voltage-clamp protocol used is indicated atop the current traces. In panel **A**, the third graph from the top is an expanded version the second graph (purple dashed box). (**B**) Time course showing effect of 10 and 30 μM SAF on the amplitude of peak *I*_Na_. Each Current amplitude (indicated with black circles) was measured at the beginning of depolarizing pulse at a rate of 2 Hz. Horizontal bar shown above indicates the SAF application. (**C**) Concentration–response curves corresponding to SAF-mediated stimulation of transient *I*_Na_ [(*I*_Na(T)_): blue filled squares] and late *I*_Na_ [*I*_Na(L)_; sustained: red open circles] in GH_3_ cells (mean ± standard error of the mean; n = 8 for each point). The current amplitude was measured at the beginning and end of a 30-ms depolarizing pulse (−100 to −10 mV). The gray smooth line indicates the goodness of fit of our model to the modified Hill equation. The EC_50_ values corresponding to the SAF-induced stimulation of *I*_Na(T)_ and *I*_Na(L)_ were 27.1 and 4.8 μM, respectively (least-squares minimization)

**Fig. 2:**
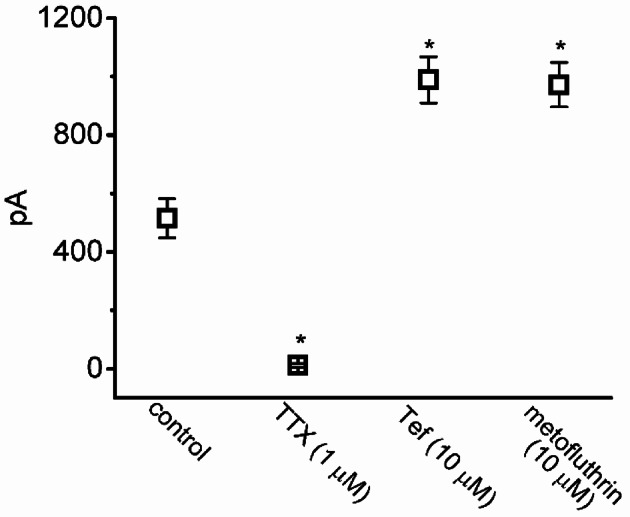
Graph showing effects of tetrodotoxin (TTX), tefluthrin (Tef) and metofluthrin on the peak amplitude of I_Na_ in GH_3_ cells. Current amplitude was measured at the beginning of each depolarizing pulse from −100 to −10 mV for a duration of 30 ms. Each point represents the mean ± standard error of the mean (n = 7). The statistical analyses were done by ANOVA-1, *P* < 0.05, followed by *post-hoc* Fisher’s least-significant different test, *P* < 0.05. ^*^Significantly different from control (*P* < 0.05)

We investigated the effects of SAF on the magnitude of I_Na_ induced by rapid membrane depolarization. The cells were bathed in Ca^2+^-free Tyrode’s solution containing 10 mM TEA and 0.5 mM CdCl_2_. TEA and CdCl_2_ were used to block K^+^ and Ca^2+^ currents, respectively. The recording pipettes were filled with a solution containing Cs^+^. As shown in Fig. 1, the tested cell was maintained at –80 mV. Subsequently, a hyperpolarizing step was applied, bringing the voltage down to –100 mV for a duration of 30 ms. This was followed by a brief depolarization step to –10 mV for 30 ms was applied to evoke I_Na_. The voltage was then returned to –50 mV for 30 ms to observe the tail current, and finally, the voltage was returned to the holding potential. Depolarizing the voltage from –100 to –10 mV over 30 ms (from a holding potential of –100 mV) robustly induced an inward current with the properties of being rapidly activated and inactivated. The rapid inward current induced by the short depolarizing pulse was identified as I_Na_ [36, 47–49] because it could be blocked by TTX (1 μM) and stimulated by either tefluthrin (Tef; 10 μM) or metofluthrin (10 μM). TTX is a potent inhibitor of I_Na_; Tef and metofluthrin effectively stimulate I_Na_ [39, 50, 51]. The results are summarized in Fig. 2.

**Fig. 3:**
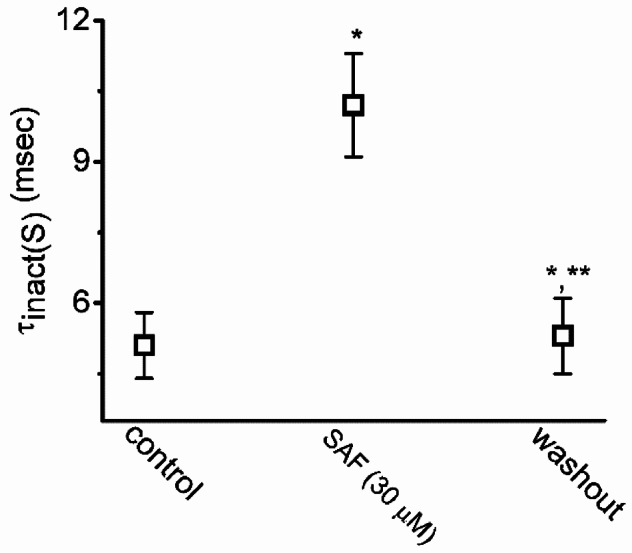
Graph showing effects of SAF on the slow component (τ_inact(S)_) in inactivation time constant of I_Na_ in GH_3_ cells. Each point represents the mean ± standard error of the mean (n = 8). The statistical analyses were done by ANOVA-1, *P* < 0.05, followed by *post-hoc* Fisher’s least-significant different test, *P* < 0.05. ^*^Significantly different from control (*P* < 0.05) and ^**^significantly different from SAF (30 μM) alone group (*P* < 0.05)

SAF exposure resulted in gradual increases in the magnitudes of *I*_Na(T)_ and *I*_Na(L)_ induced by abrupt membrane depolarization pulses (Fig. 1A). For example, 1 min after the addition of 10 and 30 μM SAF, the amplitude of *I*_Na(T)_ had increased from a control value of 512 ± 23 pA (n = 8) to 1387 ± 131 pA (n = 8, *P* < 0.05) and 1991 ± 153 pA (n = 8, *P* < 0.05), respectively; the corresponding values for *I*_Na(L)_ had increased from 18 ± 3 pA (n = 8) to 54 ± 9 pA (n = 8, *P* < 0.05) and 106 ± 17 pA (n = 8, *P* < 0.05), respectively. The amplitude of *I*_Na(L)_ was measured at the end of the depolarizing pulse from *−*100 to *−*10 mV for a duration of 30 ms. After the removal of SAF, the amplitudes of *I*_Na(T)_ and *I*_Na(L)_ returned to 523 ± 24 and 20 ± 3 pA, respectively (n = 8). SAF (30 μM) also increased the time constant τ_inact(S)_ corresponding to the slow component of *I*_Na(T)_ inactivation, with no evident change in that corresponding to the fast component. As summarized in Fig. 3, SAF (30 μM) markedly increased the τ_inact(S)_ of *I*_Na(T)_ inactivation from 5.1 ± 0.7 to 10.2 ± 1.1 ms (n = 8, *P* < 0.05). Fig. 1B illustrates the time course of stimulatory effect of SAF (10 and 30 μM) on *I*_Na_. The presence of TTX (1 μM) alone decreased *I*_Na(T)_ and *I*_Na(L)_ to 18 ± 2 pA (n = 8, *P* < 0.05) and 2 ± 1 pA (n = 8, *P* < 0.05), respectively, from a control value of 511 ± 17 pA and 32 ± 5 pA (n = 8).

SAF increased the amplitudes of *I*_Na(T)_ and *I*_Na(L)_ in a concentration-dependent manner (Fig. 1C). Using the Hill equation described in Materials and Methods, the EC_50_ values corresponding to SAF-mediated stimulation of *I*_Na(T)_ and *I*_Na(L)_ were estimated to be 27.1 ± 2.1 and 4.8 ± 0.7 μM, respectively. *I*_Na(T)_ and *I*_Na(L)_ induced by rapid depolarization pulses differentially increased in a concentration-dependent manner in GH_3_ cells.

### Effects of SAF on the steady-state I–V relationship and inactivation curve of I_Na(T)_

**Fig. 4:**
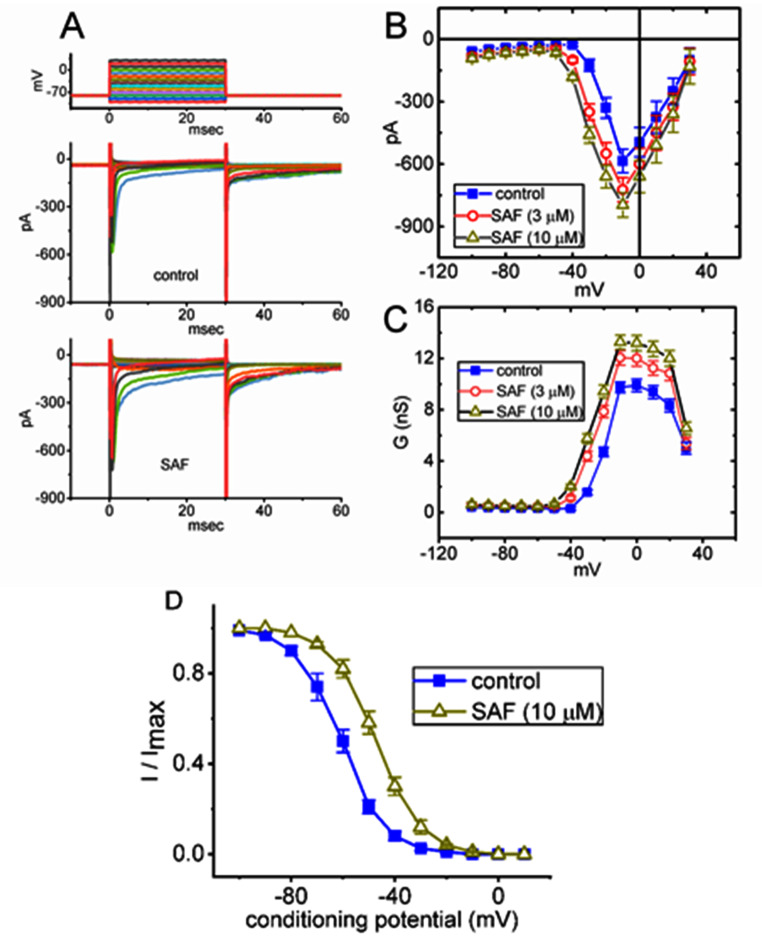
Mean current–voltage (*I-V*) or conductance-voltage relationship of *I*_Na(T)_ in GH_3_ cells. The measurements were performed as described in the legend of Fig. 1. Because we used the whole-cell mode, the test cells were maintained at −80 mV and subjected to a series of command voltages ranging from −100 to +30 mV in 10-mV increments. (**A**) Current traces during the control period (upper) and during the exposure to 3 μM SAF. The voltage-clamp protocol used is indicated atop the current traces. (**B**) Mean *I–V* relationship of *I*_Na(T)_ in the absence (blue filled squares) and with cell exposure to 3 μM SAF (red open circles) or 10 μM SAF (brown open triangles) (mean ± standard error of the mean; n = 8 for each point). *I*_Na(T)_ amplitude was measured at the beginning of each voltage pulse. Notably, the *I–V* relationship of *I*_Na(T)_ (or peak *I*_Na_) induced by 30-ms voltage pulses was shifted to more negative potentials upon SAF (3 or 10 μM) exposure. (**C**) Mean conductance versus voltage (*G-V*) of *I*_Na(T)_ in the absence (blue filled squares) and with cell exposure to 3 μM SAF (red open circles) or 10 μM (brown open triangles) (mean ± standard error of the mean; n = 8 for each point). The conductance-voltage relationship of *I*_Na(T)_ was shifted to more negative potentials during exposure to SAF. (**D**) Effect of SAF (10 μM) on the steady-state inactivation curve of *I*_Na(T)_ (mean ± standard error of the mean; n = 7 for each point). In these experiments, the conditioning voltage pulse with a duration of 30 ms to various membrane potentials between −100 and +10 mV was applied from a holding potential of −80 mV. Following each conditioning potential, a test pulse to −10 mV for a duration of 30 ms was given to activate *I*_Na_. The normalized amplitude of *I*_Na_ (*I*/*I*_max_) was constructed against the conditioning potential

To further characterize the stimulatory effects of SAF on I_Na(T)_, we investigated whether this drug perturbs the steady-state I–V relationship of I_Na(T)_ in GH_3_ cells. Fig. 4a illustrates I_Na(T)_ traces induced by different voltage steps in the presence and absence of SAF. Fig. 4B depicts the mean I–V relationship of I_Na(T)_ (i.e., V-shaped) in the absence and presence of 3 or 10 μM SAF. Fig. 4C also illustrates mean conductance versus voltage (G–V) relationship of I_Na(T)_ obtained in the control period and with the addition of 3 or 10 μM SAF. The value required for half-maximal activation voltage was found to be shifted to more negative potentials in the presence of SAF. Additionally, the steady-state inactivation curve of I_Na_ was further characterized (Fig. 4D). In these experiments, a two-step voltage-clamp protocol was applied (indicated in the legend of Fig. 4D). The results showed that cell exposure to 10 mM SAF not only increased the maximal conductance of I_Na_, but also shifted the inactivation curve to the rightward direction by approximately 14 mV with no change in the sloping factor of the curve.

### Effects of dopamine, serotonin, SAF, SAF plus dopamine, and SAF plus serotonin on I_Na(T)_ amplitude

**Fig. 5:**
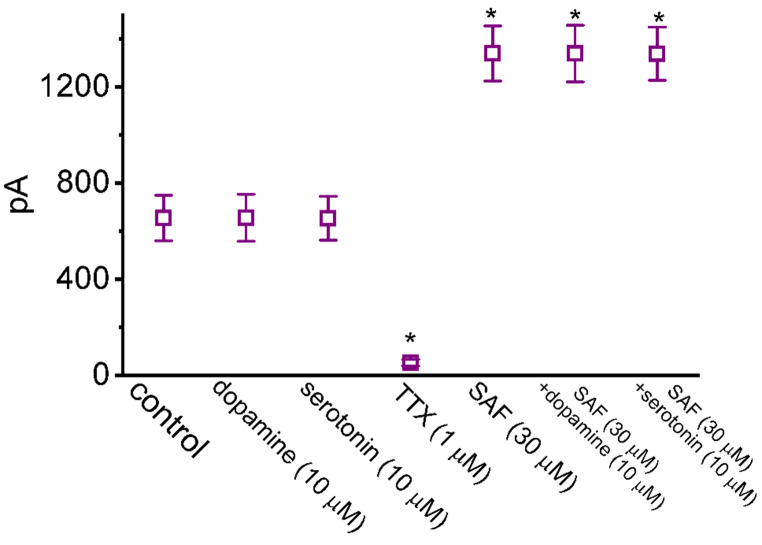
Graph illustrating the effects of dopamine, serotonin, tetrodotoxin, SAF, SAF plus dopamine, and SAF plus serotonin on the amplitude of *I*_Na(T)_ in GH_3_ cells. Each current amplitude was measured at the beginning of a short depolarizing pulse (−100 to −10 mV). In the experiments of SAF plus dopamine and SAF plus serotonin, dopamine (10 μM) or serotonin (10 μM) was added when the cells were exposed to SAF (30 μM). Each point represents the mean ± standard error of the mean (n = 7). The statistical analyses were done by ANOVA-1, *P* < 0.05, followed by *post-hoc* Fisher’s least-significant different test, *P* < 0.05. ^*^Significantly different from control (*P* < 0.05)

Studies have demonstrated the existence of MAO activity in pituitary cells [52, 53]. In the inhibition of MAO-B, the stimulatory effects of SAF on I_Na_ may result primarily from an increase in the extracellular concentration of dopamine or serotonin. We investigated whether dopamine or serotonin affects I_Na(T)_ in these cells and whether the addition of dopamine and serotonin during SAF exposure reverses the SAF-mediated increase in I_Na_. As shown in Fig. 5, the addition of neither dopamine nor serotonin altered the magnitude of I_Na(T)_; similarly, during SAF exposure, the addition of neither dopamine nor serotonin reversed the SAF-mediated increase in I_Na(T)_. Thus, under the experimental conditions employed in the present study, the SAF-mediated stimulation of I_Na(T)_ in pituitary cells may not have involved the inhibition of MAO-B activity.

### SAF-induced increase in the cumulative inhibition of I_Na(T)_ during a train of depolarizing stimuli

**Fig. 6:**
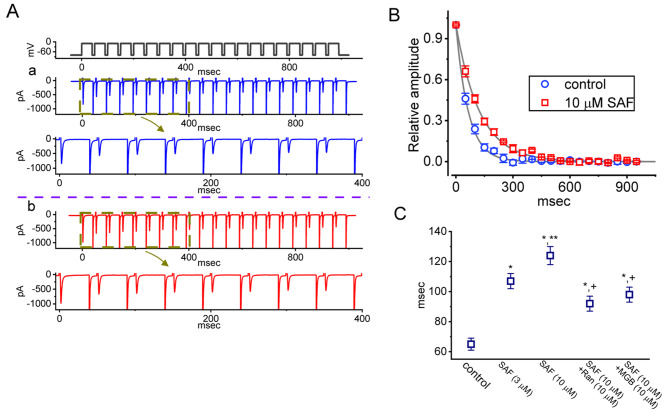
Effect of SAF on *I*_Na(T)_ decay induced by a train of depolarizing pulses in GH_3_ cells. The train of pulses comprised twenty 40-ms pulses (voltage increased to −10 mV) with 10-ms intervals at −80 mV for a total duration of 1 s. (**A**) Current traces during the control period (a, blue) and during exposure to 10 μM SAF (b, red). The voltage-clamp protocol used is indicated atop the current traces. In panel **A**, the third graphs (blue and red) from the top are the expanded forms of the second graphs (brown dashed boxes). (**B**) The relative amplitude of *I*_Na(T)_ versus pulse train duration in the absence (blue open circles) and presence (red open squares) of 10 μM SAF (mean ± standard error of the mean; n = 7 for each point). The *I*_Na(T)_ amplitudes were normalized by dividing the current amplitudes at the end of each pulse-train stimulation by those obtained at the beginning of the pulse train stimulation. The gray continuous lines on which the data points are overlaid are reliably fitted with a single exponential. (**C**) Summary graph depicting the effects of SAF (3 and 10 μM), SAF plus ranolazine (Ran), and SAF plus MGB on the decaying time constant of the current induced by a train of depolarizing command voltages ranging from –80 to –10 mV (mean ± standard error of the mean; n = 7 for each point). ^*^Significantly different from the control (*P* < 0.05), ^**^significantly different from the SAF (3 μM) alone group (*P* < 0.05), and ^+^significantly different from the SAF (10 μM) alone group (*P* < 0.05)

The inactivation of I_Na(T)_ has been demonstrated to accumulate before being elicited during repetitive short pulses [33, 34, 54]. SAF is efficacious as an add-on therapy following subthalamic nucleus deep brain stimulation in patients with Parkinson’s disease [19, 20]. Therefore, we investigated whether SAF could modify the inactivation of currents induced by a train of depolarizing stimuli. The test cells were maintained at −80 mV and subjected to repetitive depolarization to −10 mV (40 ms per pulse; rate of 20 Hz; duration of 1 s). Similar to the findings of relevant studies [34, 36], during the control period (the absence of SAF), I_Na(T)_ inactivation was noticed to be induced by 1 s of repetitive depolarization stimuli (−80 to −10 mV) with a decaying time constant of 65 ± 4 ms (n = 7; Fig. 6A, 6B and 6C). This indicated that the single-exponent process resulted in a sudden decay in the current. In the presence of 3 and 10 μM SAF, the exponential time course of I_Na(T)_ induced by the same train of depolarizing pulses was longer at 107 ± 5 ms (n = 7, *P* < 0.05) and 124 ± 6 ms (n = 7, *P* < 0.05), respectively. Furthermore, as MGB and ranolazine have been reported to suppress the amplitude of I_Na_ effectively [36, 47, 55, 56], we added ranolazine (Ran; 10 μM) and mirogabalin (MGB; 10 μM) separately in the presence of 10 μM SAF. We found they effectively attenuated the SAF-induced increase in the decaying time constant of I_Na(T)_ induced by a rapid train of pulses (Fig. 6B). The application of Ran (10 μM) or MGB (10 μM) alone decreased the decaying time constant of I_Na(T)_ during the same train of depolarizing pulses to to 39 ± 4 ms (n = 7, *P* < 0.05) or 42 ± 4 ms (n = 7, *P* < 0.05), respectively, from a control value of 66 ± 5 ms (n = 7). Thus, in addition to increasing the magnitude of I_Na(T)_, SAF prominently affects the decaying of I_Na(T)_ subjected to a 1-s train of depolarizing pulses.

### Stimulatory effects of SAF on I_Na(W)_

**Fig. 7:**
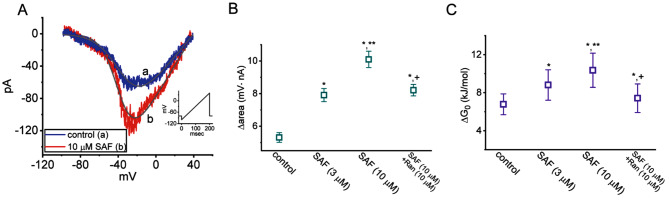
Stimulatory effects of SAF on window *I*_Na_ [*I*_Na(W)_] induced by an ascending ramp voltage *V*_ramp_ in GH_3_ cells. For the experiments, the test cells were maintained at −80 mV and subjected to *V*_ramp_ ranging from −100 to +40 mV over 200 ms (ramp speed of 0.7 mV/ms). (**A**) *I*_Na(W)_ trace in the absence (a, blue) and presence (b, red) of 10 μM SAF. The inset indicates the *V*_ramp_ protocol, and the downward deflection indicates inward-directed current. The continuous gray lines corresponding to the untreated or SAF-treated (10 μM) cells were fitted (least-squares minimization) with the Boltzmann equation. The values of *V*_1/1_ and *q* (apparent gating charge) induced by ascending *V*_ramp_ over 200 ms in the absence (blue) of SAF were, respectively, −34 mV and 2.1 *e*; the corresponding values in the presence (red) of 10 μM SAF were −38 mV and 2.8 *e*, respectively. (**B**) Summary graph illustrating the effects of SAF (3 and 10 μM) and SAF (10 μM) plus Ran (10 μM) on the Δarea of *I*_Na(W)_ in GH_3_ cells (mean ± standard error of the mean; n = 7 for each point). The value of Δarea was measured at a voltage ranging from −80 and +40 mV, which corresponded to *I*_Na(W)_ induced by the ascending V_ramp_. The statistical analyses were done by ANOVA-1, *P* < 0.05, followed by post-hoc Fisher’s least-significant different test, *P* < 0.05. ^*^Significantly different from the control (*P* < 0.05), ^**^significantly different from the SAF (3 μM) alone group (*P* < 0.05), and ^+^significantly different from the SAF (10 μM) alone group (*P* < 0.05). (**C**) Summary graph illustrating the effects of SAF (3 and 10 μM) and SAF plus Ran (10 μM) on Δ*G*_0_ (mean ± standard error of the mean; n = 7 for each point). The estimation of Δ*G*_0_ for the induction of instantaneous *I*_Na(W)_ is described in the Materials and Methods section. Notably, increasing the concentration of SAF increased Δ*G*_0_ in GH_3_ cells; further addition of Ran effectively reversed the SAF-induced increase in Δ*G*_0_. The statistical analyses were done by ANOVA-1, *P* < 0.05, followed by post-hoc Fisher’s least-significant different test, *P* < 0.05.*Significantly different from the control (*P* < 0.05), ^**^significantly different from the SAF (3 μM) alone group (*P* < 0.05), and ^+^significantly different from the SAF (10 μM) alone group (*P* < 0.05)

The induction of instantaneous I_Na(W)_ by ascending (or upsloping) V_ramp_ has been demonstrated in various excitable cells [35, 36, 38, 40, 41, 48]. In the present study, we investigated whether the addition of SAF to GH_3_ cells modulates the magnitude of I_Na(W)_ induced by ascending V_ramp_. Test cells were maintained at −80 mV and subjected to V_ramp_ ascending from −100 to +40 mV over 200 ms (i.e., ramp speed of 0.7 mV/ms) to induce I_Na(W)_ [40, 48]. The amplitude and strength (∆area) of I_Na(W)_ induced by the ascending V_ramp_ sharply increased within 1 min of SAF exposure (Fig. 7A and 7B). The ∆area values of I_Na(W)_ in the absence and presence of SAF and SAF plus Ran were calculated (Fig. 7B).

### Effect of SAF on the activation energy required for the induction of I_Na(W)_ by V_ramp_

Experimental data points corresponding to I_Na(W)_ were optimally fitted with the Boltzmann isotherm to estimate the values of q and V_1/2_ for the instantaneous I_Na(W)_ induced by V_ramp_. Using these values, the ∆G_0_ for the gating of I_Na(W)_ activation at 0 mV in the absence and presence of SAF was calculated (∆G_0_ = q × F × V_1/2_; Fig. 7C).As the SAF concentration was increased, ∆G_0_ for the induction of I_Na(W)_ by a 200-ms-long V_ramp_ also increased; the subsequent addition of Ran effectively attenuated the SAF-mediated increase in ∆G_0_. In GH_3_ cells exposed to 3 and 10 μM SAF, the ∆G_o_ values increased from a control value of 6.78 ± 1.1 to 8.81 ± 1.6 kJ/mol (n = 7, *P* < 0.05) and 10.36 ± 1.8 kJ/mol (n = 7, *P* < 0.05), respectively.

### Attenuation of the SAF-induced increase in the amplitude and Hys_(V)_ of I_Na(P)_ by MGB and Ran

**Fig. 8:**
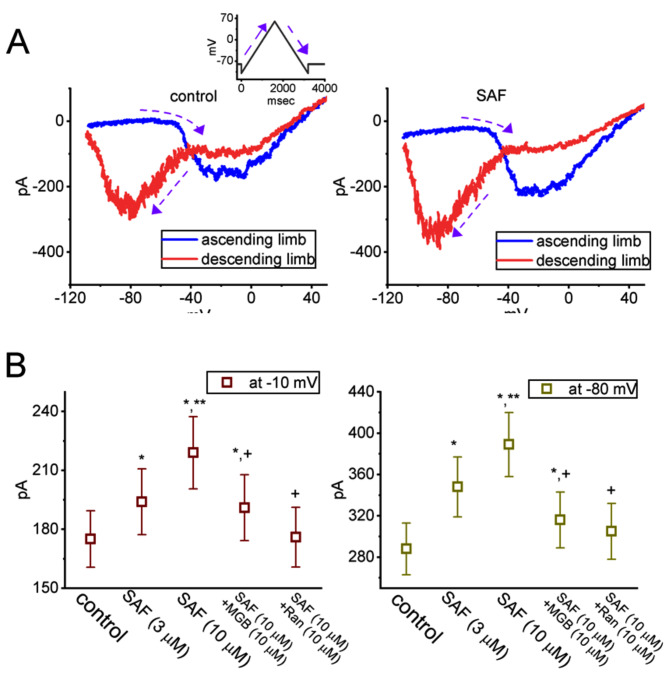
Stimulatory effects of SAF on the voltage-dependent hysteresis [Hys_(V)_] behavior of persistent *I*_Na_ [*I*_Na(P)_] induced by upright isosceles-triangular *V*_ramp_. *V*_ramp_ was supplied for 3.2 s (ramp speed of 0.1 mV/ms; digital-to-analog conversion) to induce Hys_(V)_ behavior in GH_3_ cells. (**A**) Current traces during the control period (left) and during the exposure to 10 μM SAF (right). The blue and red traces shown in each panel represent currents induced by the upsloping (ascending) and downsloping (descending) limbs of the upright isosceles-triangular *V*_ramp_, respectively. The inset indicates the voltage protocol. The dashed arrows indicate the direction of the current trajectory over time. (**B**) Summary graphs illustrating the effects of SAF (3 and 10 μM), SAF plus MGB, and SAF plus Ran on the amplitude of *V*_ramp_-induced *I*_Na(P)_ measured at −10 mV (ascending limb; left side) and −80 mV (descending limb; right side). Each point represents the mean ± standard error of the mean (n = 7). ^*^Significantly different from the control (*P* < 0.05), ^**^significantly different from the SAF (3 μM) alone group (*P* < 0.05), and ^+^significantly different from the SAF (10 μM) alone group (*P* < 0.05)

We further investigated whether SAF exposure modulated the magnitude and Hys_(V)_ behavior of I_Na(P)_ induced by an isosceles-triangular V_ramp_ in GH_3_ cells. To record whole-cell currents, the test cells were maintained at −80 mV and subjected to an upright isosceles-triangular V_ramp_ ascending from –110 and +50 mV over 3.2 s (digital-to-analog conversion; Fig. 8A). Consistent with the findings of relevant studies [43, 49, 57], we found that SAF exposure markedly increased the high and low amplitudes of I_Na(P)_ induced by the upsloping (ascending) and downsloping (descending) ends of the upright triangular V_ramp_, respectively; consequently, we observed a figure-of-eight (∞-shaped) configuration of the instantaneous I–V relationship for I_Na(P)_ and found that the configuration was enhanced by SAF. For example, when test cells were subjected to an isosceles-triangular V_ramp_ over 3.2 s (ramp speed of 0.1 mV/ms), the I_Na(P)_ amplitudes measured at −10 mV (high threshold) and −80 mV (low threshold) during the control period were 175 ± 14 pA (n = 7) and 288 ± 25 pA (n = 7), respectively. After the addition of 3 and 10 μM SAF, the I_Na(P)_ amplitude at −10 mV [high-threshold Hys_(V)_ loop] was 194 ± 17 pA (n = 7, *P* < 0.05) and 219 ± 18 pA (n = 7, *P* < 0.05), respectively; the corresponding amplitudes at −80 mV [low-threshold Hys_(V)_ loop] were 348 ± 29 pA (n = 7, *P* < 0.05) and 389 ± 31 pA (n = 7, *P* < 0.05), respectively. Adding MGB and Ran separately during SAF exposure reversed the SAF-mediated increase in the high- and low-threshold I_Na(P)_ induced by the triangular V_ramp_ (Fig. 8B). These findings indicate the unique Hys_(V)_ behavior of I_Na(P)_ induced by an isosceles-triangular V_ramp_ in GH_3_ cells; SAF exposure may increase the strength of Hys_(V)_.

### Effect of SAF on single-channel Na_V_ currents

**Fig. 9:**
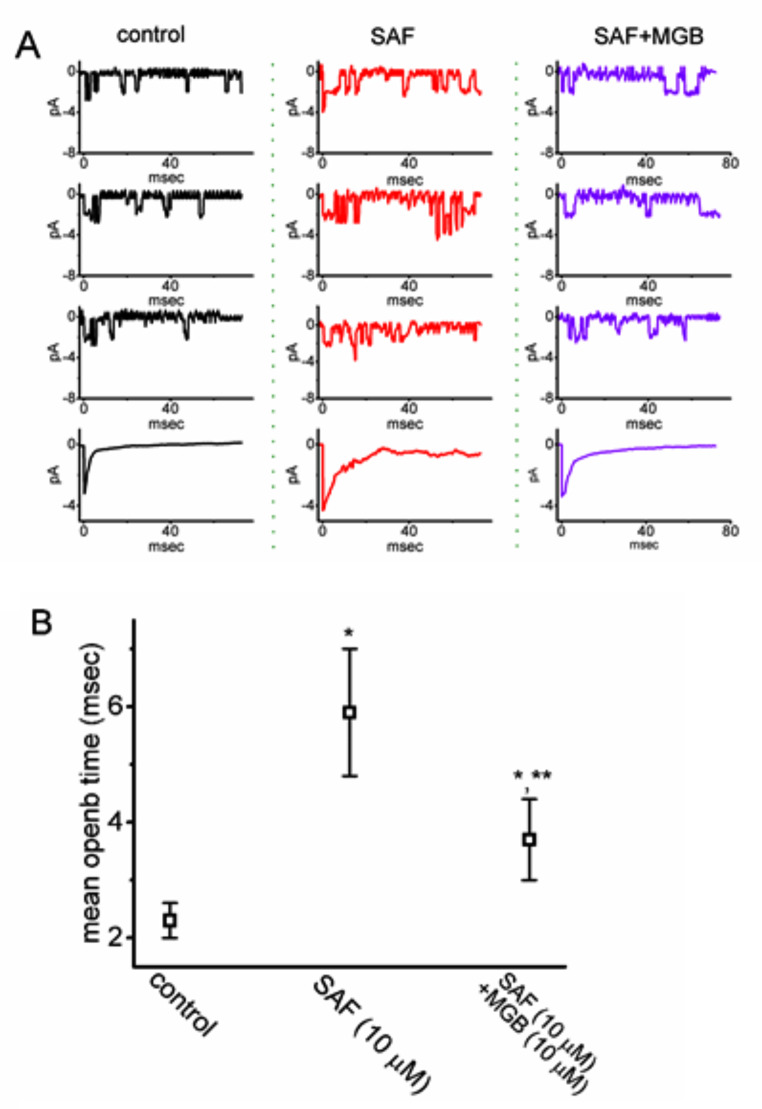
Effects of SAF on single-channel Na_V_ currents in GH_3_ cells. In (**A**), single-channel currents were induced through successive depolarizations (rate of 0.1 Hz) from a holding potential of −100 to −10 mV. Black graphs on the left indicate current traces during the control period (in the absence of SAF or SAF plus MGB); the red and blue graphs indicate the exposure to 10 μM SAF and 10 μM SAF plus 10 μM MGB, respectively. In the SAF plus MGB experiment, MGB was added 2 min after the addition of SAF. Channel opening in each record is shown as a downward deflection; the lowest traces on each side represent the average of 50 sweeps. Notably, SAF effectively increased the open-state probability of Na_V_ channels, but the subsequent addition of MGB attenuated the SAF-induced increase in the likelihood of channel opening. However, the presence of neither SAF nor MGB in addition to SAF changed the single-channel amplitude of Na_V_ channels in GH_3_ cells. (**B**) Graph showing effects of SAF, SAF plus MGB on the mean open time of Na_V_ channel (mean ± standard error of the mean; n = 7). ^*^Significantly different from control (*P* < 0.05) and ^**^significantly different from SAF (10 μM) alone group (*P* < 0.05)

To elucidate the mechanisms underlying the effects of SAF on the magnitude of I_Na_, we investigated the actions of SAF and SAF plus MGB on single-channel Na_V_ currents. This experiment was performed using the cell-attached configuration of the voltage-clamp test. Test cells were placed in K^+^-rich solution, and the recording pipette was filled with Na^+^-rich solution. SAF (10 μM) increased channel activity and decelerated current inactivation when the cells were exposed to depolarization stimuli ascending from –100 to –10 mV (rate of 0.1 Hz; Fig. 9A). Furthermore, the addition of MGB (10 μM) in the presence of 10 μM SAF reduced the probability of channel opening. The addition of 10 μM SAF markedly increased channel activity from 0.027 ± 0.007 to 0.091 ± 0.006 (n = 7, *P* < 0.05); the addition of 10 μM MGB in the presence of SAF reduced the open-state probability of the channel to 0.041 ± 0.007 (n = 7, *P* < 0.05). Moreover, with the presence of 10 μM SAF, the mean open time of Na_V_ channels was prolonged to 6.5 ± 0.7 (n = 7, *P* < 0.05) msec from a control value of 2.3 ± 0.3 msec (n = 7). However, no considerable modifications were noted in the amplitude of the single-channel current in the presence of SAF or SAF plus MGB (control, 2.01 ± 0.35 pA; SAF, 2.03 ± 0.37 pA; SAF plus MGB, 2.00 ± 0.44 pA; n = 7; *P* > 0.05). Consistent with these findings, the mean open time of the Na_V_ channel in the presence of 10 μM SAF (5.9 ± 1.1 ms; n = 7; *P* < 0.05) was longer than that in the control period (2.3 ± 0.3 ms, n = 7); the subsequent further addition of 10 μM MGB decreased the mean open time to 3.7 ± 0.7 ms (n = 7, *P* < 0.05) (Fig. 9B). Although SAF did not change the amplitude of single-channel currents, it enhanced channel activity and decelerated inactivation of Na_V_-channel opening in GH_3_ cells. MGB added during SAF exposure reversed the SAF-induced increase in Na_V_ channel activity.

### Docking prediction of SAF on human MAO_B and Na_V_ channel

**Fig. 10:**
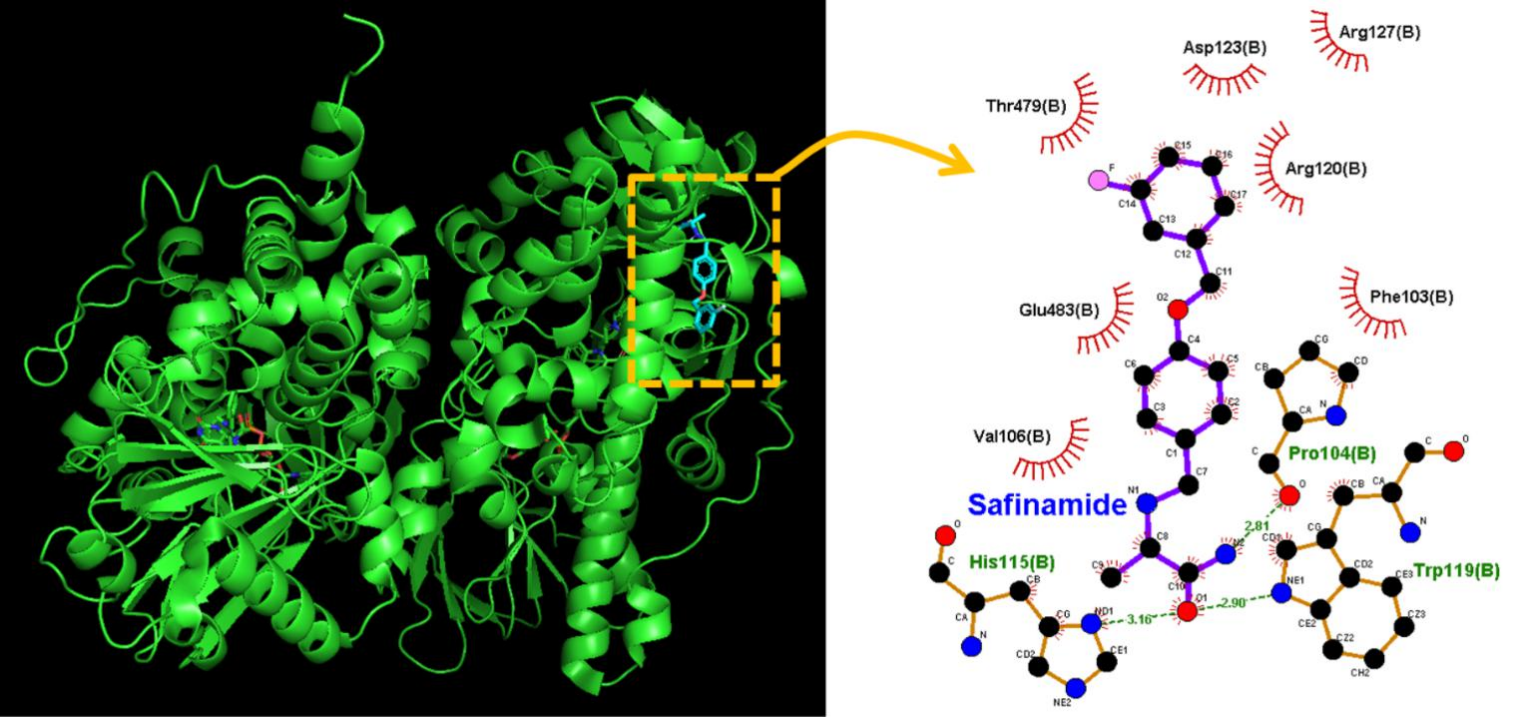
Predicted docking interactions between SAF and monoamine oxidase B (MAO-B). The protein structure was obtained from the Protein Data Bank (ID: 1GOS); the chemical structure of SAF was obtained from PubChem [compound CID: 131682 (3D conformer)]. MAO-B was docked with SAF (yellow dashed box on the left) through PyRx, and the corresponding interaction diagram was generated using LigPlot^+^. The red arcs with spokes radiating toward the ligand (SAF) indicate hydrophobic interactions between SAF and MAO-B. The green dotted line indicates the hydrogen bond between SAF and Pro 104, His 115, or Trp 119, with the corresponding bond lengths being 2.81, 3.16, or 2.90 Å

Using PyRx, we further explored the molecular docking between human MAO-B (structure: https://www.rcsb.org/structure/1GOS) and SAF. Figure 10 illustrates the predicted binding sites of SAF. SAF engages in hydrophobic interactions with certain amino acid residues, such as Phe 103, Val 106, Arg 120, Asp 123, Arg 127, Thr 479, and Glu 483. In addition to interacting with intramolecular hydrogen bonds [58], SAF forms three hydrogen bonds with the Na_V_-channel residues Pro 104, His 115, and Trp 119, with the bond lengths being 2.81, 3.16, and 2.90 Å, respectively. The binding affinity for the interaction between SAF and MAO-B is –7.8 kcal/mol, and the upper and lower root-mean-square deviations (RMSD) in atomic positions were 49.76 and 60.68, respectively. In line with the findings of relevant studies [21, 23, 59], we observed that the interaction between MAO-B and SAF resulted in a substantial decrease in MAO-B activity.

**Fig. 11:**
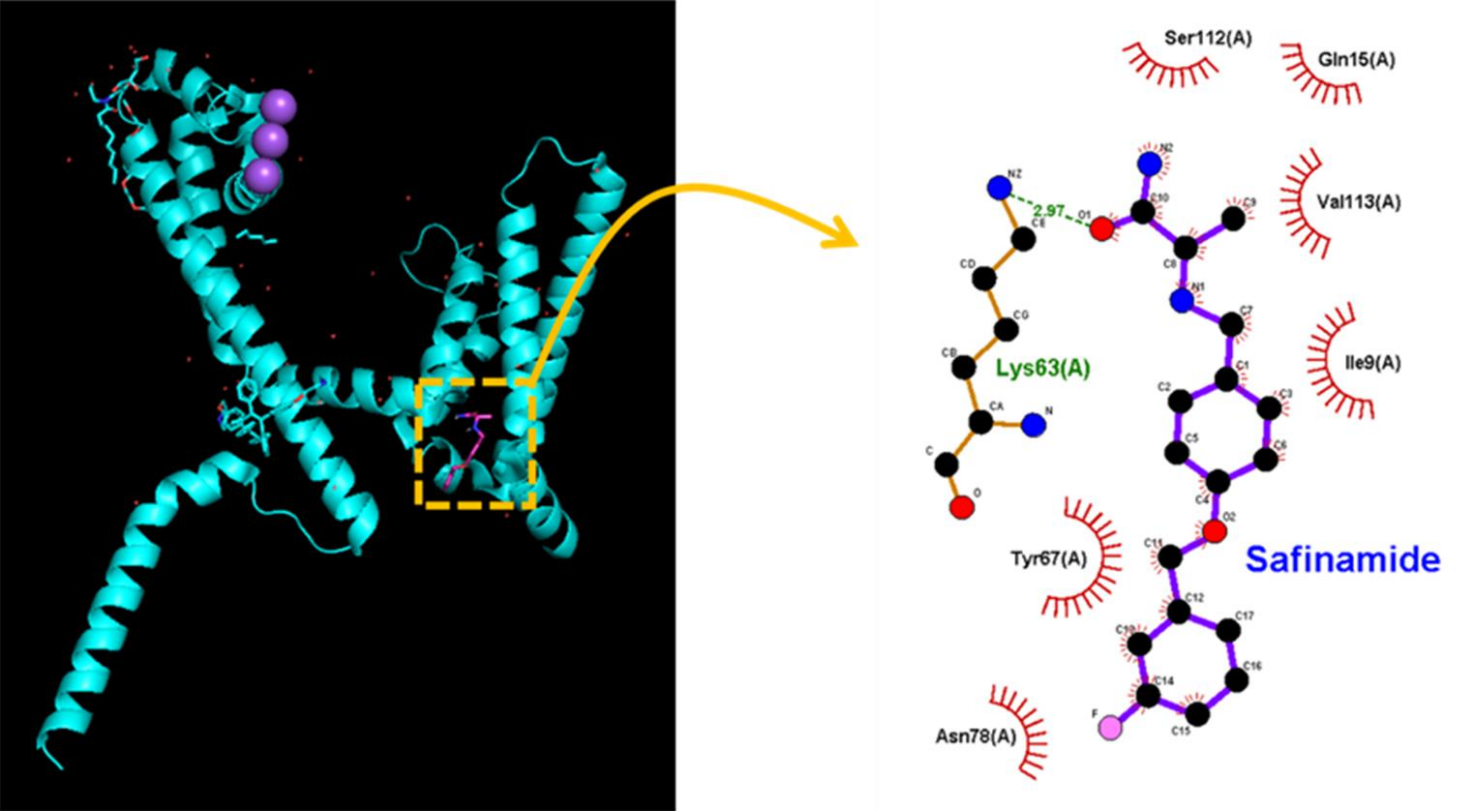
Predicted docking interactions between Na_V_ channels and SAF. The protein structure of a Na_V_ channel was obtained from the Protein Data Bank (ID: 6Z8C), and the chemical structure of SAF was obtained from PubChem [compound CID: 131682 (3D conformer)]. A Na_V_ channel was docked with SAF (yellow dashed box on the left) using PyRx; the corresponding interaction diagram was generated using LigPlot^+^. In the image on the right, the red arcs with spokes radiating toward the ligand (SAF) indicate hydrophobic interactions between SAF and several amino acid residues, whereas the green dashed line indicates the hydrogen bond between SAF and Lys 63, the length of which was 2.97 Å. The docking regions appear to be adjacent to the transmembrane region (position: residues 82–102) and the membrane segment (position: residues 46–67). The interactions probably alter structural constraints, thereby increasing the open-state probability of Na_V_ channels

We further explored the molecular docking between Na_V_ channels and SAF. Figure 11 and Supplementary Fig. 1 depicts the predicted binding sites of SAF. After docking, SAF forms a hydrogen bond with Lys 63, with the bond length being 2.97 Å. SAF further engages in hydrophobic interactions with several residues, including Ile 9, Gln 15, Tyr 67, Asn 78, Ser 112, and Val 113. The binding affinity for the interaction between SAF and a Na_V_ channel was found to be −6.8 kcal/mol, and the upper and lower RMSD values were 23.00 and 25.59, respectively. The affinity energy was close to the estimated ∆*G*_0_ for the induction of *I*_Na(W)_ by *V*_ramp_ in the presence of SAF. Thus, SAF can dock with both MAO-B and Na_V_ channels, thereby presumably reducing structural constraints and increasing channel activity. Collectively, the dual effects of SAF on MAO-B and Na_V_ channel activities [23] may considerably affect the functional activities and thus may be beneficial in the treatment of various neurological disorders [11, 15, 25, 29]. However, since a prokaryotic Na_V_ channel (i.e., Na_V_M) was used in this prediction, whether SAF can modulate the function of Na_V_M as observed in GH3-cells’ NaV channel needs to be further examined.

## Discussion

Our key findings are as follows. SAF stimulated *I*_Na_ in a concentration-, time-, and frequency-dependent manner. It differentially stimulated *I*_Na(T)_ and *I*_Na(L)_ induced by short depolarizing pulses. SAF increased the time constant of the decay of *I*_Na(T)_ induced by a train of depolarizing pulses but increased the strength and ∆*G*_0_ of *V*_ramp_-induced *I*_Na(W)._ The Hys_(V)_ strength of *I*_Na(P)_ (in both low- and high-threshold loops) was greater when the cells were exposed to an upright isosceles-triangular *V*_ramp_. Cell-attached single-channel current recordings revealed a SAF-induced increase in the open-state probability of the channel without any change in the single-channel amplitude. Molecular docking between SAF and both MAO-B and Na_V_ channels indicated the existence of similar structural motifs, which facilitate SAF binding to MAO-B and Na_V_ channels. SAF may reach the binding site once the Na_V_ channel protein is highly activated and is in its open state or conformation. Overall, these findings suggest that SAF-mediated modulation of the magnitude, gating, and Hys_(V)_ behavior of *I*_Na_ may be independent and upstream of its inhibitory action on MAO-B activity.

As mentioned, SAF inhibits the activity of MAO-B [2, 14, 23, 24, 52, 53, 60]. Thus, in the present study, the stimulation of *I*_Na_ by SAF was expected to be associated with the inhibition of MAO-B by SAF and with subsequent increases in the concentrations of dopamine and serotonin. However, the exposure of cells to dopamine and serotonin did not lead to any changes in the magnitude of *I*_Na(T)_ induced by rapid membrane depolarization pulses. Adding dopamine and serotonin separately to the bath solution in the presence of SAF exerted no effects on the SAF-stimulated *I*_Na(T)_. Therefore, the stimulatory effects of SAF on *I*_Na(T)_ and *I*_Na(L)_ may be mediated by a mechanism other than that involving the inhibition of MAO-B activity.

The time-dependent decrease in *I*_Na(T)_ induced by a 20-Hz train of depolarizing pulses (40-ms pulses of voltage ascending from –80 to –10 mV; rate of 20 Hz; duration of 1 s) was decelerated by SAF. The results indicate that there is use dependence of *I*_Na(T)_ during repetitive depolarization, as demonstrated previously [34–36, 60]. SAF may lead to progressive gain-of-function changes by altering and decelerating the inactivation of currents. Thus, the SAF-mediated increase in *I*_Na(T)_ may be closely associated with use-dependent attenuation of the magnitude of *I*_Na(T)_ induced by a train of depolarizing pulse stimuli.

We further estimated the ∆area and ∆*G*_0_ values for the instantaneous *I*_Na(W)_ induced by an ascending *V*_ramp_, both of which were found to be markedly higher when SAF was present. Adding Ran in the presence of SAF reversed the SAF-mediated increase in the ∆area and ∆*G*_0_ of the current. Because the magnitude of *I*_Na(W)_ is primarily responsible for the background (steady state) conductance of Na^+^ and the electrical firing of excitable cells [37, 38, 41, 61–63], SAF may increase the firing frequency of action potentials by enhancing the strength of *I*_Na(W)_. It is important to note that the currents elicited by V_ramp_ may also result from late/persistent *I*_Na_ and slow closed-state inactivation. Whether the SAF-mediated augmentation of ramp currents was caused by increasing *I*_Na(W)_ still needs to be investigated. Moreover, how the activation energy of V_ramp_-induced *I*_Na(W)_ can be increased in the SAF presence remains to be further studied.

We observed the nonlinear voltage-dependent Hys_(V)_ behavior of *I*_Na(P)_ during the control period and the exposure of the cells to SAF, SAF plus MGB, or SAF plus Ran [36, 42]. The Hys_(S)_ behavior was induced by exposing the cells to an upright isosceles-triangular *V*_ramp_ for 3.2 s. SAF increased the peak of *I*_Na(P)_ induced by the ascending (upsloping) limb of the triangular *V*_ramp_, particularly at –10 mV, and the amplitude of *I*_Na(P)_ induced by the descending (downsloping) end of *V*_ramp_, particularly at –80 mV. Thus, we noted a figure-of-eight (∞-shaped) configuration of the Hys_(V)_ loop of current induced by the triangular *V*_ramp_; the strength of this behavior was discovered to be considerably enhanced in the presence of SAF. Thus, *V*_ramp_ induced two distinct types of *I*_Na(P)_: high-threshold current and low-threshold current. The high-threshold *I*_Na(P)_ was excessively induced [at a voltage range where peak *I*_Na(T)_ was induced maximally] by the upsloping limb of the triangular *V*_ramp_; by contrast, the low-threshold *I*_Na(P)_ was induced by the downsloping end of the triangular *V*_ramp_. Furthermore, the trajectories of currents induced by the ascending and descending limbs of the triangular *V*_ramp_ followed the counterclockwise and clockwise directions, respectively. Adding MGB and Ran separately in the presence of SAF reduced the SAF-mediated increases in high- and low-threshold *I*_Na(P)_ induced by *V*_ramp_. It also needs to be noted that the increase of the current at –80 mV in the descending limb of the voltage protocol might be caused by the ability of SAF to activate the persistent *I*_Na_. Thus, whether SAF affect the Na_V_-channel deactivation still warrants further investigations.

It needs to be noted that as the concentration of extracellular Ca^2+^ was decreased, the gating of *I*_Na_ might be altered [64]. However, the presence of extracellular Ca^2+^ was also allowed to activate voltage-gated Ca^2+^ current and then to interfere with the examination of voltage-gated Na^+^ current. Therefore, whether safinamide affects the amplitude and gating of *I*_Na_ when the concentration of external Ca^2+^ is within the physiological range remains to be further studied.

The cell-attached current recordings revealed that SAF increased the open-state probability of Na_V_; this increase was reversed by the subsequent addition of MGB. Furthermore, SAF extended the mean open time of single Na_V_ channels; however, this effect of SAF was reversed by MGB. SAF did not modify the amplitude of single Na_V_ channels; this indicates that the interaction between SAF and Na_V_ channels is secondary to alterations, which possibly occur at a location remote from the pore region of the channels.

Pharmacokinetic studies have reported that the maximal plasma concentrations of SAF after single 2.5, 5.0, and 10.0 mg/kg doses can be approximately 1000, 2500, and 5500 ng/mL (or 3.3, 8.3 and 18.2 μM), respectively [65]. The EC_50_ values for the SAF-mediated stimulation of *I*_Na(T)_ and *I*_Na(L)_ were 27.1 ± 2.1 and 4.8 ± 0.7 μM, respectively; this indicates that the stimulatory effects of SAF on *I*_Na_ are within the clinical therapeutic range, although the SAF concentration in cerebrospinal fluid is still unclear [60]. The IC_50_ for the SAF-mediated inhibition of MAO-B activity has been reported to be approximately 30–40 nM [23]. Notably, the magnitude of SAF-stimulated *I*_Na_ depends strongly on various factors, including the pre-existing resting potential, the firing pattern of the action potential, the concentration of SAF in cells, and a combination of the aforementioned factors. Regarding the inhibitory effect of SAF on MAO-B, SAF may lyse and remove the components of the surface membrane of various host cells (Edmondson and Binda, 2018), thus accessing cytosolic or mitochondrial enzymes. Thus, the stimulatory perturbation of *I*_Na_ [*I*_Na(T)_, *I*_Na(L)_, *I*_Na(W)_, and *I*_Na(P)_] is clinically achievable by using SAF and may have pharmacological, therapeutic, and toxicological relevance in humans [12, 16, 17].

SAF may increase blood pressure. Na_V_ channels are functionally distributed across vascular smooth muscle cells [66–68]. The mRNA transcripts of the α subunits of Na_V_1.1, Na_V_1.2, Na_V_1.3, and Na_V_1.6, together with those of the β1 and β3 subunits, have been detected in GH_3_ cells [69]. The extent to which SAF-induced hypertensive events [4, 10] are associated with the stimulatory effects of SAF on *I*_Na_ in vascular smooth muscle cells (i.e., Na_V_1.7), heart cells (i.e., Na_V_1.5 or Na_V_1.6), and skeletal muscle cells (i.e., Na_V_1.4) is worth investigation.

In contrast to our findings, earlier studies showed SAF might suppress *I*_Na_ magnitude in different preparations [21, 26–29]. It will be important to determine whether the inhibitory effect of SAF on *I*_Na_ is associated with either the decrease of the activity of monoamine oxidase or the production of reactive oxygen species [13, 14, 22–25]. Of note, although SAF was considered a potential anticonvulsant based on prior reports of *I*_Na_ attenuating property, clinically, the anticonvulsive activity of SAF was not proven, as only open-label studies comparing with baseline were provided [70, 71]. The inhibitor of Na_V_ channels could generally be considered as an anticonvulsant, however, despite the unclear underlying ionic mechanism, the stimulator of Na_V_ channels virtually might not become a pro-epileptic drug, because of a wide range of epileptic disorders through which the initiation or epileptogenesis is largely unclear. Furthermore, the difference on the effect of *I*_Na_ may be the result of dissimilar channel isoforms, expression levels of isoforms, the species, the auxiliary proteins in the cell types, and the different concentrations of compounds used for each cell type. Direct comparisons of sodium channel kinetic properties were thus restricted to data within the same cell type. Nevertheless, it is likely that the stimulatory effect of SAF on *I*_Na_ is preferentially linked to its bindings to Na_V_1.5 and/or Na_V_1.6 isoforms of the channel. Further characterization and interpretation of the modulatory effect of SAF on *I*_Na_ and overall cellular excitability in different cell types or network should be implemented.

### Electronic supplementary material

Below is the link to the electronic supplementary material.

Supplementary Material 1

## Data Availability

The datasets used and/or analyzed during the current study are available from the corresponding author on reasonable request.
