# Supplementary material for: Safinamide, an inhibitor of monoamine oxidase, modulates the magnitude, gating, and hysteresis of sodium ion current
Source: BMC Pharmacol Toxicol. 2024 Feb 8;25:17. doi: 10.1186/s40360-024-00739-5 (PMC10851555; doi:10.1186/s40360-024-00739-5)
Supplement: Supplementary file 1 — Supplementary Material 1 [file 40360_2024_739_MOESM1_ESM.docx]

**Supplementary Information**

In this work, we further investigated how the protein of Na_V_ channel could be delicately docked with tefluthrin (Tef) with PyRx software. Tef, a type I pyrethroid insecticide, is known to activate *I*_Na_ (Wu et al., 2009, 2015). The predicted binding sites of the Tef molecule are illustrated in **Supplementary Figure 1**. Of interest, as being presumably docked to Na_V_ channel, Tef can form hydrogen bond with residue Asn 78 at a distance of 3.26 Å. Moreover, the Tef molecule can form hydrophobic contacts with several residues, including Ile 9, Phe 14, Gln 15, Glu 59, Tyr 67, Trp 77, Ser 112, Arg 118. The results showed that Tef could interact with the amino-acid residues of Na_V_ channel with a binding affinity of -7.5 kcal/mol, and the values of upper and lower RMSD were estimated to be 2.37 and 5.45, respectively. The binding affinity for the docking by Tef appeared to be greater than that for the Saf docking. As a results, like the docking of Saf (**Figure 9**), the Tef molecule can interact with certain residues in Na_V_ channels, thereby presumably decreasing the structural constraints and causing an increase in channel activity.


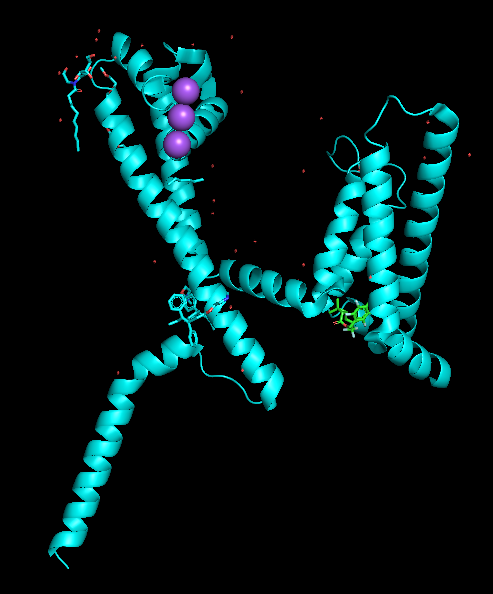

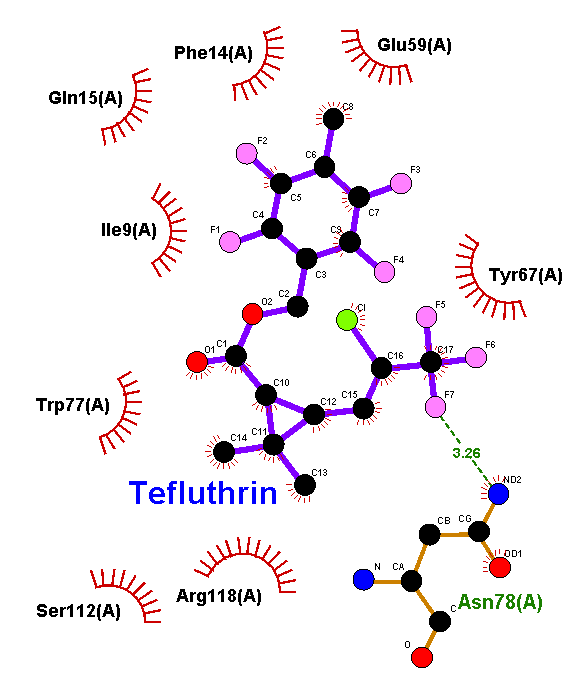


**Supplementary Figure 1.** Predicted docking results demonstrating the interactions between Na_V_ channel and tefluthrin (Tef). Protein structure of Na_V_ (SCN) channel was derived from protein data bank (PDB, ID: 6Z8C). The structure of Na_V_ channel was noticeably docked with the Tef molecule (yellow dashed box at the center of the left side) through PyRx, while the diagram showing interaction between Na_V_ channel and the Tef molecule was generated from LigPlot^+^. Of note, at the right side, the red arcs with spokes that radiate toward the ligand (i.e., Tef) shows the hydrophobic contacts with several amino acid residues, whereas the green dashed line shows the formation of hydrogen bond with the residue Asn 78 at a distance of 3.26 Å. The docking regions appear to be adjacent to transmembrane region (i.e., position: 82-102) and membrane segment (i.e., 46-67). As a result, it is likely that, similar to the docking results of safinamide (SAF) shown in **Figure 9**, such interactions change the structural constraints, thereby causing an increase in the open-state probability of the channel.

**References**

Wu SN, So EC, Liao YK, Huang YM. Reversal by ranolazine of doxorubicin-induced prolongation in the inactivation of late sodium current in rat dorsal root ganglion neurons. Pain Med 2015;16(5):1032-1034.

Wu SN, Wu YH, Chen BS, Lo YC, Liu YC. Underlying mechanism of actions of tefluthrin, a pyrethroid insecticide, on voltage-gated ion currents and on action currents in pituitary tumor (GH_3_) cells and GnRH-secreting (GT1-7) neurons. Toxicology 2009;258(1):70-77.
